# Pain management in patients with long-bone fractures in a district hospital in KwaZulu-Natal, South Africa

**DOI:** 10.4102/phcfm.v7i1.818

**Published:** 2015-12-02

**Authors:** Adeleye M. Awolola, Laura Campbell, Andrew Ross

**Affiliations:** 1 Department of Family Medicine, University of KwaZulu-Natal, South Africa

## Abstract

**Background:**

This study reviewed pain severity and assessment as recalled by patients with long-bone fractures. The focus was on the intervals between admission, pain assessment and analgesic provision, as delay of analgesia for acute pain can result in complex chronic pain syndromes.

**Aim:**

The aims were to explore patients’ recollection of pain severity and assessment in an emergency department (ED) and whether analgesia prescribed in the ED correlated with pain severity.

**Setting:**

The study site was a district hospital ED in KwaZulu-Natal, South Africa.

**Methods:**

This exploratory study considered aspects of pain in adults with long-bone fracture who were admitted to an ED and later referred to an orthopaedic unit. Data collection took place in the orthopaedic unit where participants were requested to recall their pain severity (using a visual analogue scale) whilst in the ED.

**Results:**

Ninety-three patients participated, most of whom were African males. Over 60% recalled their pain severity in the ED as 5 or greater on a visual analogue perception scale. No formal tool was used to assess or record pain in the ED, and there was no association between recalled pain severity and type of analgesia prescribed.

**Conclusion:**

The majority of patients were assessed for pain in the ED. Analgesia given to most patients was inadequate for the degree of pain they experienced. A pain assessment protocol should be developed for doctors and nurses to serve as a guideline in assessing patients with long-bone fractures and prescribing appropriate analgesia.

## Abstrait

**Analyse de la gestion de la douleur chez les patients atteints de fractures des os longs dans un hôpital de district dans le KwaZulu-Natal en Afrique du Sud**.

**Contexte:**

Cette étude a examiné la sévérité et l’évaluation de la douleur telles que rapportées a posteriori par des patients atteints de fractures des os longs. L'accent était mis sur les intervalles entre l'admission, l’évaluation de la douleur et l'administration d'analgésique, le retard de l'analgésie en cas de douleur aiguë pouvant entrainer des syndromes complexes de douleur chronique.

**But:**

Les objectifs étaient d'analyser le souvenir de la sévérité et de l’évaluation de la douleur des patients dans un service d'urgence (SU), et si l'analgésie prescrite dans le SU correspondait à la sévérité de la douleur.

**Cadre:**

Le site d’étude était le SU d'un hôpital de district dans le KwaZulu-Natal en Afrique du Sud.

**Méthodes:**

Cette étude exploratoire a examiné les aspects de la douleur chez les adultes atteints de fracture des os longs admis dans un SU et plus tard envoyés vers une unité orthopédique. La collecte des données s'est faite au sein de l'unité orthopédique, où les participants ont été invités à se remémorer la sévérité de la douleur (en utilisant une échelle visuelle analogique - VAS) lors de leur passage au SU.

**Résultats:**

Quatre-vingt treize patients ont participé, dont la plupart étaient des hommes africains. Plus de 60% se sont rappelé d'une sévérité de la douleur au service d'urgence de 5 ou plus sur une échelle de perception de type EVA. Aucun outil formel n'a été utilisé pour évaluer ou enregistrer la douleur au SU, et il n'existait aucun lien entre le souvenir de la sévérité de la douleur et le type d'analgésie prescrit.

**Conclusion:**

La majorité des patients ont été évalués quant à la gestion de la douleur au sein du SU. L'analgésie administrée à la plupart des patients était insuffisante par rapport au degré de douleur qu'ils avaient subi. Un protocole d’évaluation de la douleur doit être développé pour les médecins et les infirmières afin de servir de ligne directrice en termes d’évaluation de l’état des patients atteints de fractures des os longs et de prescription d'une analgésie appropriée.

## Introduction

Internationally many patients with acute pain following trauma do not receive any form of analgesia on admission to a hospital emergency department (ED).^1^ As an example, research carried out in the United States of America assessed analgesia administered to patients with acute trauma in an ED and reported that half received no analgesia at discharge.^1^ Two-thirds waited for up to one hour before receiving any analgesia, and a third received inappropriate analgesia in relation to pain severity. It is particularly concerning that analgesia was not administered in the ED, even to patients with clearly documented evidence of moderate to severe pain.^1^

It is pertinent to review pain in patients with bone fractures in South Africa, as the incidence of bone fractures is high. It has been estimated that a road traffic collision occurs every four seconds in South Africa, and the resultant trauma leads to multiple injuries, including fractures.^2^ EDs in South Africa are generally underprepared for their workload, and improvements could be made, including management of pain.^3^ This study grew from a concern that patients in KwaZulu-Natal, South Africa who were referred to an orthopaedic unit from an ED appeared to have unmanaged pain.

With regard to pain following a fracture, a large study of patients with extremities fractures reported that only 64% received any analgesia, and analgesic management did not form part of ED protocols.^4^ A paediatric ED study indicated that only a third of children with acute, severe pain due to long-bone fractures received any analgesia.^5^

Under-treatment of acute pain is of concern, because if pain remains untreated a patient may develop a complex pattern of chronic pain and disability.^6^ This chronic pain results from ‘central sensitisation’ where the nervous system develops a process of ‘wind-up’ and becomes regulated to a persistent state of high reactivity.^6^ This persistent state maintains pain, even after the initial injury might be healed. Acute pain following acute trauma such as bone fractures has been demonstrated to induce central sensitisation.^7^ Although the time between fracture pain and central sensitisation has not been determined, it would seem necessary to treat any acute pain as soon as possible. Most importantly, prompt and appropriate pain management is essential as a fundamental human right.^8^

An extensive literature search found no literature on pain management of patients with long-bone fracture in a South African ED setting, and hence this study will add to the information available on this important topic. The aim of this study was to assess patients’ perceptions of pain severity following a long-bone fracture and to review their perceptions of how this pain was assessed in an ED. Associations between recalled pain severity and analgesia that was prescribed were explored. An ED presents a unique opportunity for prompt and appropriate management of any acute pain.

## Research methods and design

The design was an exploratory cross-sectional study, and the setting was a district hospital (DH) in KwaZulu-Natal which serves a large community of over 220 000 people. Approximately 50 patients with long-bone fractures are reviewed every month in the ED. For the purpose of this study long-bone fractures were considered to be fractures of the humerus, ulna, radius, femur, tibia and fibula. All patients with long-bone fractures are assessed and stabilised in the ED, and only those requiring admission with multiple open, severely displaced and severely painful fractures are referred onwards for specialist care in an orthopaedic unit. Approximately 40 patients with long-bone fractures are referred from the ED to the orthopaedic unit each month. This study population was chosen because pain in this group of patients is usually severe, and there is a need for prompt, appropriate management in the ED to prevent long-term complications.

As this was an exploratory study, a sample size of 100 was chosen as expedient. Inclusion criteria included adult patients with acute long-bone fractures who were referred from an ED to the orthopaedic unit. The following patients were excluded: those with associated pain which may not be related to long-bone fracture (for example, bone cancer), those who presented to the ED more than one week post-fracture, and those who were unable to give consent.

Every third patient admitted to the orthopaedic unit who met the inclusion criteria was recruited over the study period. Data were collected using two methods: an interview between the patient and the researcher, which was carried out in the orthopaedic unit, and a hospital chart review. The interviews took place at various times after admission to the orthopaedic unit, and patients were requested to recall their stay in the ED. They were asked to recall the following: their maximum pain severity in the ED, whether someone assessed their pain in the ED, and their estimations of intervals between admission and pain assessment and analgesia administration.

Pain management is recognised as a fundamental human right, and at the time of interview the researcher (A.M.A.), who is a medical doctor, assessed pain using a visual analogue scale (VAS) in the orthopaedic unit and immediately prescribed appropriate analgesia if required.

Participants were requested to score the severity of their recalled pain in the ED on the VAS. The VAS was considered to be easy to use in a clinical setting and has been validated in diverse cultural and linguistic settings.^9^ The VAS has been found to be useful in measuring pain in bone fractures in a range of injuries, including spinal fractures.^10^

Data on analgesia prescribed in the ED were obtained from patients and patient chart review. Data were captured on a Microsoft Excel spreadsheet and analysed descriptively. Associations between recalled pain severity and analgesia prescribed in the ED were explored using the Chi-square test and analysis of variance (ANOVA).

## Ethical considerations

Permission to conduct the study was obtained from the Research Ethics Committee at the University of KwaZulu-Natal (Ref. BE 023/13), the Provincial Department of Health and hospital managers.

## Results

**FIGURE 1:**
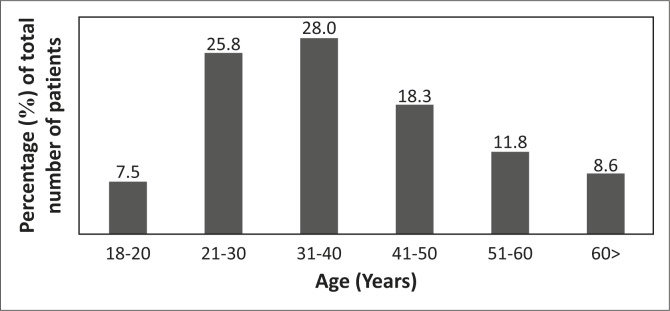
Age distribution of participants.

Two hundred and seventy-five patients were admitted to the orthopaedic unit over an eight-month period from January to August 2014, and 93 patients were interviewed as part of this study. Ages of the participants ranged from 18 to over 60 years, and over half (56.0%) were in the 30–50-years age group. The majority were male (84.0%) and most were black African (94.0%), with 3.0% of participants being white and 3.0% from other racial groups. The age distribution is represented diagrammatically in Figure 1.

**FIGURE 2:**
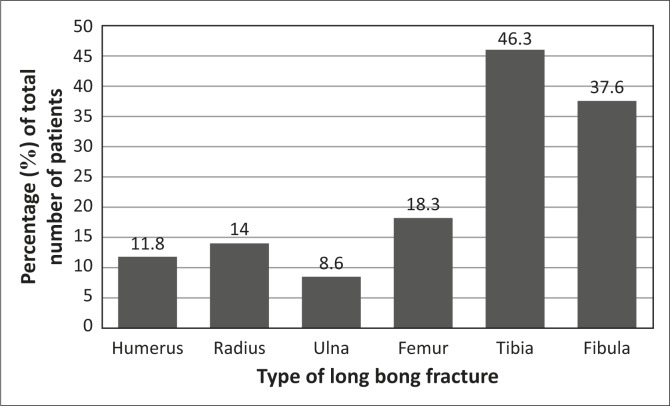
Site of long-bone fracture.

Half of the fractures occurred in the leg (51.6%). Some patients had more than one type of fracture; for instance, 31.2% had both tibia and fibula fractures. The fracture sites are represented in Figure 2.

**FIGURE 3:**
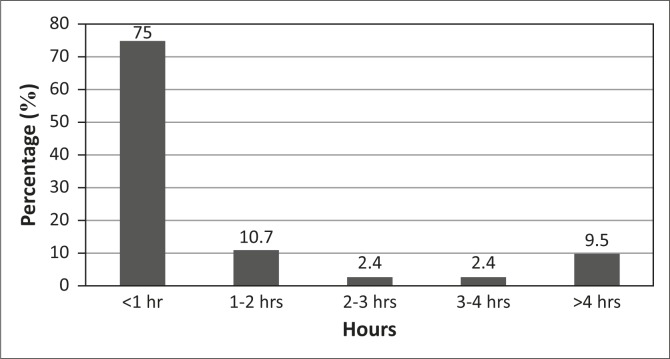
Perceived intervals between emergency department admission and pain assessment.

Most participants (62.4%) were interviewed by the researcher more than 48 hours following admission to the ED. The majority (96.0%) perceived that they had some form of pain assessment in the ED, and these participants recalled being only verbally questioned about pain with no formal pain evaluation scale used. Two per cent of the participants recalled that they were not asked about pain intensity. Most (82.0%) recalled receiving an initial pain assessment from a doctor only and 18.0% received an initial pain assessment from a nurse only. The perceived intervals between ED admission and pain assessment are summarised in Figure 3.

**FIGURE 4:**
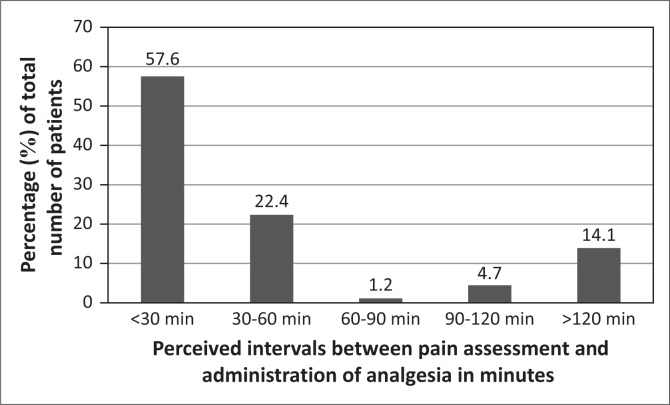
Perceptions of interval between pain assessment and administration of analgesia.

Participants’ reports concerning the interval between pain assessment and administration of analgesia are illustrated in Figure 4.

**FIGURE 5:**
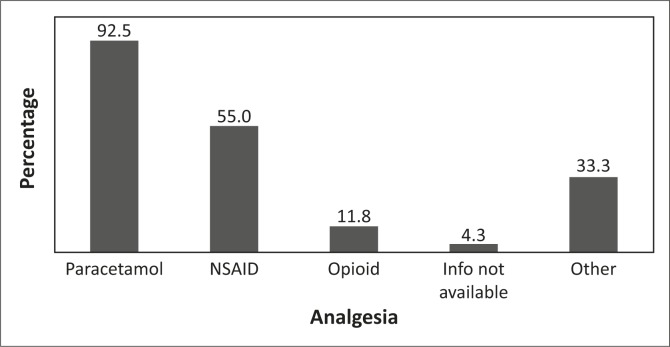
Types of analgesia recorded during emergency department stay.

A chart record review of the time spent in the ED revealed that half of the analgesia prescribed was administered orally (46.0%) and half via injection (54.0%). The analgesia varied from a weak class to a strong class (as described by the World Health Organization).^11^ Some patients received more than one class of analgesia. Some patients (4.3%) reported receiving analgesia but information on the specific type was not recorded in the patient's chart (information not available). Some patients (33.3%) received opioid-like agents such as tramadol and/or paracetamol with codeine (classified by the researcher as ‘other analgesia’ in Figure 5). Paracetamol was administered orally whilst nonsteroidal anti-inflammatory agents (NSAIDs) were administered either orally or via intramuscular (IM) injections. Opioids used for analgesia were given via IM or intravenous injection. The types of analgesia recorded during ED stay are shown in Figure 5.

Most of the participants (64.0%) recalled their pain as being at level 5 or greater. The range of recalled VAS pain scores is summarised in Table 1.

**TABLE 1:** Recalled pain score on the visual analogue scale.

Recalled pain score reported on VAS	%respondents
1	4.3
2	7.5
3	10.8
4	10.8
5	32.3
6	10.8
7	11.6
8	7.3
9	2.2
10	2.2

ANOVA showed no association between type of analgesia provided in the ED and participants’ recalled pain score on the VAS (*F* = 1.001, and *p* = 0.396).

## Discussion

The aim of the study was to explore participants’ recall of pain they experienced whilst in the ED and to assess intervals between admission, assessment and receipt of analgesia. Importantly the study also considered whether ED analgesia correlated with pain severity. Participants had severe or complex fractures, and review of pain assessment and treatment is vital as a lack of early pain management intervention can lead to complications such as chronic pain syndromes.

Results indicated that more males that females were referred to the orthopaedic unit and participants reported significant levels of pain whilst in the ED. Participants were mainly reviewed by doctors in the ED and the intervals between admission, assessment and receipt of analgesia varied between participants. This suggests that no standardised systems were in place for assessment of pain and delivery of analgesia.

The study methods were primarily descriptive, however analysis using ANOVA was found to be useful to hone in on the lack of correlation between reported pain severity and type/World Health Organization class of analgesia prescribed. Each of the findings will be discussed in more detail below.

More men were admitted than women. Although chronic pain syndromes tend to be more common in women, it is important not to neglect the prompt management of acute pain in men.^6^ Most participants were in the age group 30–50 years, and a relatively young population may have a higher predisposition to central sensitisation resulting in chronic pain.^12^ Further study could consider other demographic features which could predispose to central sensitisation, including not being employed, low educational status and smoking.^6^

In this study the majority of participants were assessed for pain by a doctor rather than by a nurse. This is an interesting finding, as in South Africa there are generally more nurses than doctors available in an ED setting. South African literature advocates that nurses should become more involved with triage of patients in EDs.^13^ The potential assessment of pain by nurses requires further exploration in this context.

Although most participants recalled that their pain was assessed within an hour of admission to the ED, the time between assessment and analgesia availability varied and was prolonged (over two hours) in some instances. This finding has major implications in terms of human rights issues, and can also potentially induce chronic pain syndrome. The literature supports that if nurses became more involved, gaps between admission, assessment and analgesia administration may be shortened.^14^

It was of concern that no tool was used to measure or record pain in the ED. This is similar to findings of a Tanzanian study on pain in an ED setting.^15^ The introduction of a nurse-initiated triage of pain could potentially improve assessment of pain, using a standardised, validated pain assessment tool. Such an assessment tool could be supported by a pain assessment protocol to serve as a guideline in prescribing appropriate analgesia.

Despite patients rating their pain score as 5 or more, the two most commonly prescribed analgesics were oral paracetamol and an NSAID, which according to the World Health Organization are not adequate for moderate to severe pain.^11^ This finding is similar to that of another ED-based study, which showed that an NSAID was the commonest analgesia used for patients with fractures, and was usually administered as a once-off IM dose.^15^ Reasons why relatively mild analgesia was prescribed despite patients reporting moderate to severe pain, and how effective mild analgesia is in this setting require further investigation.

## Limitations of the study

A limitation to this study is that the recall of data was used. Patients were asked to recall events such as severity of pain, who assessed the pain, and the interval between admission, assessment and analgesic administration, and their recall may have been impaired or distorted as they were in an acutely stressful situation. An alternative data collection method using a point prevalence of pain in an ED or continuous assessment of patients from admission to discharge may permit the gathering of more reliable data.

## Conclusion and recommendations

It was encouraging that the majority of patients were assessed for pain and given analgesia in the ED. However, it was of concern that the majority was assessed by doctors, and the potential role of the ED nurse in pain management requires further exploration. As an example, ensuring cooperation and collaboration between nurses and doctors may require attention; a complication may arise if a nurse assesses a pain as significant and a doctor prescribes a mild analgesia.

There appeared to be no standardised methods to assess and record pain, and analgesia prescriptions did not seem to be appropriate for the severity of the pain. In addition, there were prolonged periods between pain assessment and analgesia provision.

A pain assessment protocol should be developed for doctors and nurses to serve as a guideline in assessing patients with long-bone fractures and prescribing appropriate analgesia. This protocol could also provide an opportunity for continuous re-evaluation and assessment of patients in a bid to ensure that no opportunity is missed to provide adequate analgesia. Further research is required in this important area to guide the training and practice of healthcare professionals.
